# 15-deoxy-**Δ_12,14_**-prostaglandin J**_2_** Down-Regulates Activin-Induced Activin Receptor, Smad, and Cytokines Expression via Suppression of NF-*****κ*****B and MAPK Signaling in HepG2 Cells

**DOI:** 10.1155/2013/751261

**Published:** 2013-09-24

**Authors:** Seung-Won Park, Chunghee Cho, Byung-Nam Cho, Youngchul Kim, Tae Won Goo, Young Il Kim

**Affiliations:** ^1^Department of Biotechnology, Catholic University of Daegu, Daegu 712-702, Republic of Korea; ^2^School of Life Science, Gwangju Institute of Science and Technology (GIST), Gwangju 500-712, Republic of Korea; ^3^Department of Life Sciences, The Catholic University of Korea, Bucheon 420-743, Republic of Korea; ^4^Department of Internal Medicine, College of Korean Medicine, Kyung Hee University, Seoul 130-872, Republic of Korea; ^5^Department of Agricultural Biology, National Academy of Agricultural Science, RDA, Suwon 441-100, Republic of Korea; ^6^Medical Science Research Institute, Kyung Hee University Medical Center, Seoul 130-872, Republic of Korea; ^7^East-West Medical Research Institute, Kyung Hee University, Seoul 130-872, Republic of Korea

## Abstract

15-Deoxy-Δ^12,14^-prostaglandin J_2_ (15d-PGJ_2_) and activin are implicated in the control of apoptosis, cell proliferation, and inflammation in cells. We examined both the mechanism by which 15d-PGJ_2_ regulates the transcription of activin-induced activin receptors (ActR) and Smads in HepG2 cells and the involvement of the nuclear factor-**κ**B (NF-**κ**B) and mitogen-activated protein kinase (MAPK) pathways in this regulation. Activin A (25 ng/mL) inhibited HepG2 cell proliferation, whereas 15d-PGJ_2_ (2 **μ**M and 5 **μ**M) had no effect. Activin A and 15d-PGJ_2_ showed different regulatory effects on ActR and Smad expression, NF-**κ**B p65 activity and MEK/ERK phosphorylation, whereas they both decreased IL-6 production and increased IL-8 production. When co-stimulated with 15d-PGJ_2_ and activin, 15d-PGJ_2_ inhibited the activin-induced increases in ActR and Smad expression, and decreased activin-induced IL-6 production. However, it increased activin-induced IL-8 production. In addition, 15d-PGJ_2_ inhibited activin-induced NF-**κ**B p65 activity and activin-induced MEK/ERK phosphorylation. These results suggest that 15d-PGJ_2_ suppresses activin-induced ActR and Smad expression, down-regulates IL-6 production, and up-regulates IL-8 production via suppression of NF-**κ**B and MAPK signaling pathway in HepG2 cells. Regulation of ActR and Smad transcript expression and cytokine production involves NF-**κ**B and the MAPK pathway via interaction with 15d-PGJ_2_/activin/Smad signaling.

## 1. Introduction

 Activins are either heterodimers or homodimers of inhibin *β* subunits (*β*A*β*A, *β*B*β*B, or *β*A*β*B) [1]. The biological activities of activins are mediated by receptor complexes that consist of 2 different activin serine/threonine kinase receptors (ActR), type I (ActR I) and type II (ActR II) [2]. Smad2 and Smad3 proteins are phosphorylated by specific activated type I serine/threonine kinase receptors. Formation of dimeric complexes leads to phosphorylation of Smad2 and Smad3, subsequent complex formation with Smad4, and regulation of activin-responsive genes [3, 4]. Smad7 functions as an inhibitor of transforming growth factor-*β* (TGF-*β*) family signaling, including activin signaling [5, 6]. Activin-responsive genes have been implicated in the control of homeostasis, development, proliferation, apoptosis, differentiation, and inflammation in diverse cellular systems [2].

15-Deoxy-Δ^12,14^-prostaglandin J_2_ (15d-PGJ_2_) is a derivative of prostaglandin D_2_ and is a natural ligand of peroxisome proliferator-activated receptor-gamma (PPAR*γ*), which is a transcriptional nuclear receptor [7, 8]. 15d-PGJ_2_ has a broad spectrum of biological effects including apoptosis, cell proliferation, inflammation, and induction of antioxidant enzyme expression [9]. Recent studies demonstrated that PPAR*γ* agonists prevent TGF-*β*1/Smad3 signaling in human hepatic stellate cells [10]. 15d-PGJ_2_ inhibits the expression of TGF-*β*-induced connective tissue growth factor by inhibiting Smad2 phosphorylation, which is independent of PPAR; 15d-PGJ_2_ might also act through a PPAR-dependent mechanism in human hepatoma cells. In addition, 15d-PGJ_2_ might prevent liver fibrosis that is induced by environmental toxins [11]. Reports on the effect of TGF-*β* signaling on NF-*κ*B activity in cancer cells are conflicting. TGF-*β* has been reported to decrease NF-*κ*B activation in both salivary gland cells and breast cancer cells through a mechanism that involves the increased expression of I*κ*B-*α* [12, 13]. TGF-*β* treatment has also been shown to have no effect on NF-*κ*B activation in vulvar carcinoma cells [14]. 15d-PGJ_2_-mediated up-regulation of interleukin-8 (IL-8) is secondary to the activation of the mitogen-activated protein kinase (MAPK) signaling pathway and inhibition of the NF-*κ*B signaling pathway [15]. However, the effects of PPAR*γ* on activin signaling in HepG2 cells have not been determined. We examined both the mechanism by which 15d-PGJ_2_ regulates the transcription of activin-induced activin receptors and Smads in HepG2 cells and how the NF-*κ*B and MAPK pathways are involved in this regulation.

## 2. Materials and Methods

### 2.1. Materials

Recombinant human activin A was purchased from R&D Systems (Minneapolis, MN, USA) and 15-Deoxy-Δ^12,14^-prostaglandin J_2_ was obtained from Cayman Chemical (Ann Arbor, MI, USA).

### 2.2. Cell Culturing

The human hepatoma cell line HepG2 was obtained from the American Type Culture Collection (Rockville, MD, USA). Cells were cultured in Dulbecco's modified Eagle's medium that contained 10% fetal bovine serum (Gibco-BRL, Grand Island, NY, USA) and antibiotics, at 37°C in a humidified atmosphere that contained 5% CO_2_ and 95% air. 

### 2.3. MTT Assay

Cell proliferation was measured with CellTiter 96 Aqueous One Solution (Promega, Madison, WI, USA). Cells were seeded at 1 × 10^4^ cells/well in 96 well plates and then incubated with activin A and 15d-PGJ_2_ for 72 h. Cell viability was determined by using a colorimetric assay with PMS/MTS solution. The absorbance was measured at 492 nm and background subtraction was carried out at 650 nm.

### 2.4. RNA Extraction and Real-Time PCR Analysis

 Total RNA was isolated from cultured cells by using RNA-Bee solution kit (Tel-Test, Friendswood, TX, USA). First strand cDNA synthesis was performed with 1 *μ*g of total RNA and transcribed to cDNA by using a reverse transcription system with random hexamers (Promega) according to the manufacturer's protocol. The primer sequences and product size were as follows: ActR IA forward 5′-GATGAGAAGTCATGGTTCAGG-3′, reverse 5′-TATGTTTGGCCTTTGTTGATC-3′, 651 bp; ActR IB forward 5′-CTGGCTGTCCGTCATGATGCA-3′, reverse 5′-CAATTCGCTCTCAGAGTCTCC-3′, 683 bp; ActR IIA forward 5′-ACCAGTGTTGATGTGGATCTT-3′, reverse 5′-TACAGGTCCATCTGCAGCAGT-3′, 456 bp; ActR IIB forward 5′-TTCTGCTGCTGTGAAGGCAAC-3′, reverse 5′-GAGGTCGCTCCTCAGCAATAC-3′, 699 bp; Smad2 forward 5′-TAGGTGGGGAAGTTTTTGCT-3′, reverse 5′-TTTTCATGGGACTTGATTGG-3′, 411 bp; Smad3 forward 5′-GGGCTCCCTCATGTCATCTA-3′, reverse 5′-GGCTCGCAGTAGGTAACTGG-3′, 443 bp; Smad4 forward 5′-CCCAGGATCAGTAGGTGGAA-3′, reverse 5′-CCATGCCTGACAAGTTCTGA-3′, 452 bp; Smad7 forward 5′-TCCTGCTGTGCAAAGTGTTC-3′, reverse 5′-TTGTTGTCCGAATTGAGCTG-3′, 447 bp; *β*-actin forward 5′-CTTCTACAATGAGCTGCGTG-3′, reverse 5′-TCATGAGGTAGTCAGTCAGG-3′, 305 bp. Real-time PCR was performed on a Chromo4 Detector real-time system (Bio-Rad, Hercules, CA, USA) with SsoFast EvaGreen Supermix (Bio-Rad). PCRs were performed with 2 *µ*L of cDNA in 20 *µ*L reaction mixtures that consisted of 10 *µ*L of the SsoFast EvaGreen Supermix, 2 *μ*L of primers, and 6 *µ*L of PCR grade water. The reactions were performed with a denaturation step at 95°C for 30 s followed by 45 cycles of 95°C for 5 s and 55°C to 62°C for 12 s. The crossing point of activin receptor or Smad with *β*-actin was calculated by using the formula 2(^−(target gene-*β*actin)^), and the relative amounts were quantified.

### 2.5. IL-6 and IL-8 Enzyme-Linked Immunosorbent Assay

 The concentrations of IL-6 and IL-8 in the harvested cell culture supernatant were determined by enzyme-linked immunosorbent assay (ELISA) according to the manufacturer's instructions (R&D Systems).

### 2.6. NF-*κ*B p65 Activity Assay

 At each time point, the culture medium was removed and the cells were scraped into 2 mL of cold phosphate-buffered saline (PBS) with phosphatase inhibitors. The cell suspension was centrifuged (500 rpm for 5 min at 4°C). The cell pellets were lysed in 25 *µ*L of complete lysis buffer (Active Motif, Carlsbad, CA, USA) for 30 min on ice on a rocking platform that was set at 150 rpm. The extract was centrifuged (14,000 rpm for 10 min at 4°C) and the supernatant was used for activity assays following the kit protocol. The protein concentration in the nuclear extract was determined by using the BCA protein assay (Pierce, Rockford, IL, USA). An ELISA for NF-*κ*B p65 activity was performed according to the manufacturer's instructions (Active Motif).

### 2.7. Immunoblot Analysis

Cells were plated at 2 × 10^6^ cells in a 100 mm tissue culture dish. The following day, they were treated with drugs in a concentration-dependent manner. After treatment, the cells were washed with cold PBS and lysed in the tissue culture dish by using lysis buffer [20 mM Tris-HCl (pH 7.5), 150 mM NaCl, 1 mM Na_2_EDTA, 1 mM EGTA, 1% Triton, 2.5  mM sodium pyrophosphate, 1 mM *β*-glycerophosphate, 1 mM Na_3_VO_4_, and 1 *μ*g/mL leupeptin] that contained 1 mM phenylmethylsulfonyl fluoride. The protein concentration was determined by using the BCA protein assay. Proteins (30 *μ*g) were fractionated by performing SDS-PAGE on a 12% gel and transferred by performing electrophoresis onto a nitrocellulose membrane. The membranes were blocked with 5% nonfat dry milk for 1 h at room temperature and incubated with anti-ERK1, antiphospho-ERK1/2 (Thr 202), anti-MEK-1/2, antiphospho-MEK-1/2 (Ser 218/Ser 222), or *β*-actin antibodies (Santa Cruz Biotechnology, Santa Cruz, CA, USA) at a 1 : 250 dilution in Tris-buffered saline that contained 0.05% tween-20 (TBS-T) for 1 h. Then, after washing with TBS-T for 1 h, the membranes were reacted with horseradish peroxidase-conjugated secondary antibody that had been diluted to 1 : 2,500 withTBS-T for 1 h at room temperature. After washing the membranes with TBS-T for 1 h, proteins were detected by using an ECL Chemiluminescence Kit (Santa Cruz Biotechnology). Protein expression was analyzed by using a Chemiluminescence Imaging System (Davinch-Chemi, Seoul, Korea). Protein band densities were quantified by employing the new wave module of the Image Solution Program (IMT i-Solution, Inc., Vancouver, BC, Canada).

### 2.8. Statistical Analyses

The experiments were repeated for three independent samples, for which the values are expressed as the mean ± SEM. All comparisons were analyzed by using the nonparametric Kruskal-Wallis one-way analysis of variance test. Values of *P < 0.05 were deemed to indicate a statistical significance.

## 3. Results

### 3.1. Effects of Activin and 15d-PGJ_2_ on HepG2 Cell Proliferation

**Figure 1:**
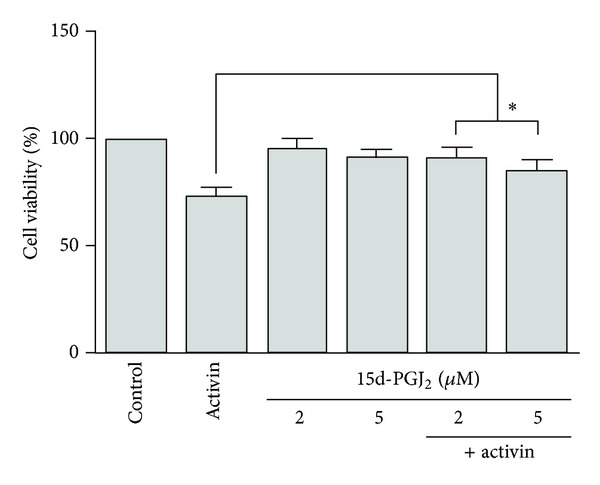
Effects of activin and 15-Deoxy-Δ^12,14^-prostaglandin J_2_ (15d-PGJ_2_) on HepG2 cell proliferation. HepG2 cells were exposed to activin A (25 ng/mL) or 15d-PGJ_2_ (2 or 5 **μ**M) alone or in combination for 72 h. Cell viability was determined by using an MTT assay. The data are shown as the mean ± SEM of triplicate samples. *P < 0.05, comparison of stimulation by 15d-PGJ_2_ and activin A versus activin A stimulation alone.

HepG2 cells were treated with activin A or 15d-PGJ_2_. Cell proliferation was determined by using an MTT assay. Activin A suppressed cell proliferation, whereas 15d-PGJ_2_ had no effect. Stimulation of the cells with both activin A and 15d-PGJ_2_ enhanced HepG2 cell proliferation compared to activin A stimulation alone (Figure 1).

### 3.2. 15d-PGJ_2_ Regulates Activin-Induced Activin Receptor and Smad mRNA Expression in HepG2 Cells

**Figure 2:**
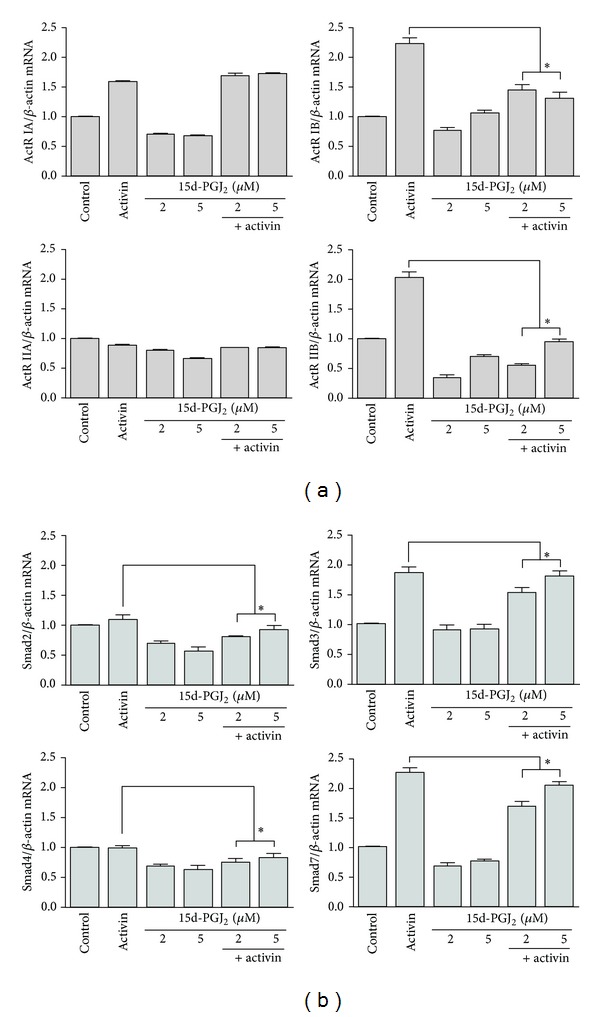
Activin receptor and Smad mRNA expression in HepG2 cells. HepG2 cells were treated with activin A (25 ng/mL) and 15d-PGJ_2_ (2 or 5 **μ**M) for 72 h, and levels of ActR (a) and Smad (b) mRNA were measured by real-time PCR. The crossing point of activin receptors with *β*-actin was entered into the formula, 2^−(target gene-*β* actin)^, and the relative amounts were quantified. The data represent the mean ± SEM of 3 independent samples. *P < 0.05, comparison of stimulation with 15d-PGJ_2_ and activin A versus activin A stimulation alone.

To determine the effect of 15d-PGJ_2_ on the expression of ActRs and Smads in HepG2 cells in the presence of activin A, HepG2 cells were treated with activin A and 15d-PGJ_2_ for 72 h. We performed real-time PCR to determine ActR and Smad mRNA expression. Activin A increased the expression of ActR IA, IB, IIB, Smad3, and Smad7 mRNA compared to the control, whereas ActR IIA mRNA expression decreased and Smad2 and 4 mRNA expression remained unchanged. 15d-PGJ_2_ inhibited both ActR and Smad mRNA expression at both 2 *μ*M and 5 *μ*M compared to the control. When the cells were stimulated with both 15d-PGJ_2_ and activin A, 15d-PGJ_2_ suppressed the activin-induced increase in ActR and Smad mRNA expression, but increased ActR IA mRNA levels compared to activin A stimulation alone (Figures 2(a) and 2(b)).

### 3.3. 15d-PGJ_2_ Down-Regulates Activin-Induced IL-6 Production and Up-Regulates Activin-Induced IL-8 Production in HepG2 Cells

**Figure 3:**
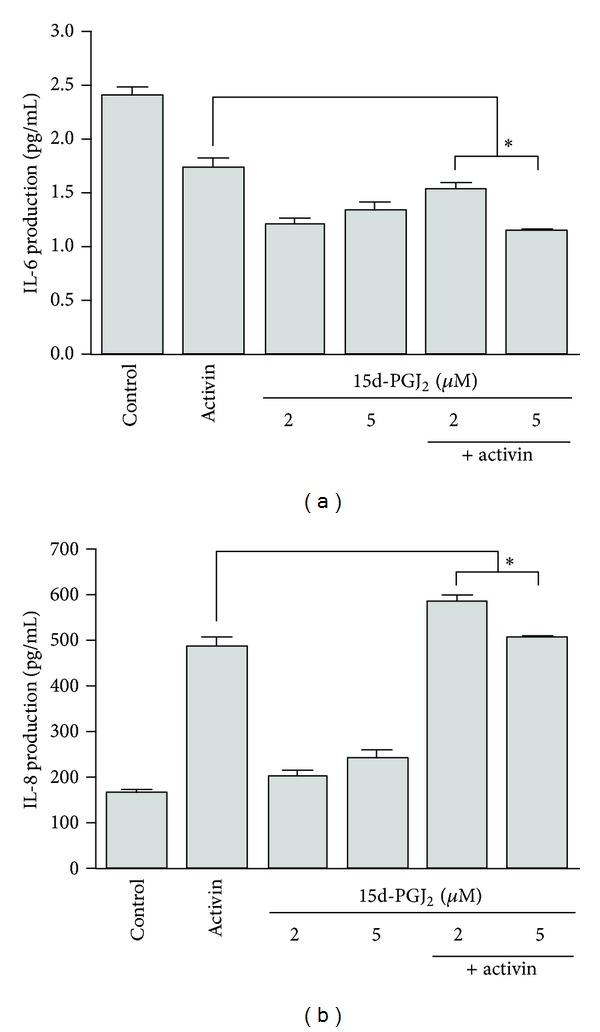
IL-6 and IL-8 production in HepG2 cells. Cells were stimulated with activin A (25 ng/mL) and 15d-PGJ_2_ (2 or 5 **μ**M) for 72 h. The amounts of IL-6 (a) and IL-8 (b) in the cell culture supernatants were determined using ELISA. The data represent the mean ± SEM of 3 independent samples. *P < 0.05, comparison of stimulation with 15d-PGJ_2_ and activin A versus activin A stimulation alone.

HepG2 cells were treated with activin A and 15d-PGJ_2_ for 72 h, and then IL-6 and IL-8 levels in the cell culture supernatant were measured by using an ELISA. Activin A and 15d-PGJ_2_ both suppressed IL-6 production compared to the control. When the cells were stimulated with both 15d-PGJ_2_ and activin A, 15d-PGJ_2_ decreased activin-induced IL-6 production compared to activin A stimulation alone (Figure 3(a)). Activin A and 15d-PGJ_2_ increased IL-8 production compared to the control. When the HepG2 cells were stimulated with both 15d-PGJ_2_ and activin A, 15d-PGJ_2_ enhanced activin-induced IL-8 production compared to activin A stimulation alone (Figure 3(b)). IL-1*β* production was not observed after individual stimulation or costimulation (data not shown).

### 3.4. 15d-PGJ_2_ Inhibits Activin-Induced NF-*κ*B p65 Activity in HepG2 Cells

**Figure 4:**
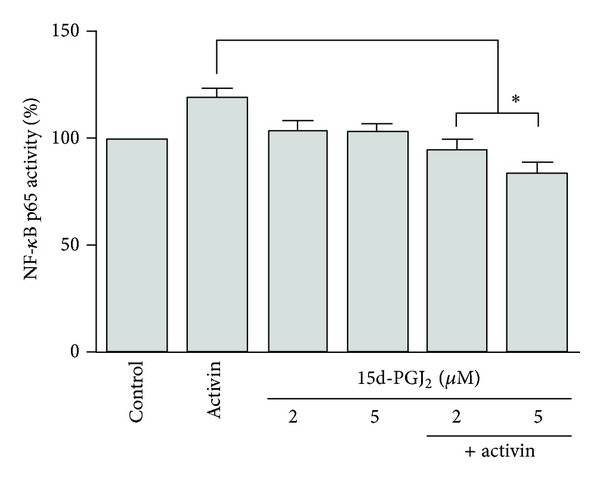
NF-*κ*B p65 activity in HepG2 cells. Cells were stimulated with activin A (25 ng/mL) and 15d-PGJ_2_ (2 or 5 *μ*M) for 72 h. NF-*κ*B p65 activity in the culture nuclear extracts was determined using ELISA. The data represent the mean ± SEM of 3 independent samples. *P < 0.05, comparison of stimulation with 15d-PGJ_2_ and activin A versus activin A stimulation alone.

HepG2 cells were treated with activin A and 15d-PGJ_2_ for 72 h and then the NF-*κ*B p65 activity of the nuclear fraction was measured using ELISA. Activin A increased NF-*κ*B p65 activity compared to the control, whereas 15d-PGJ_2_ did not affect NF-*κ*B p65 activity. When cells were stimulated with both 15d-PGJ_2_ and activin A, 15d-PGJ_2_ inhibited the activin-induced NF-*κ*B p65 activity compared to activin A stimulation alone (Figure 4).

### 3.5. 15d-PGJ_2_ Suppresses Activin-Induced MEK/ERK Activation in HepG2 Cells

**Figure 5:**
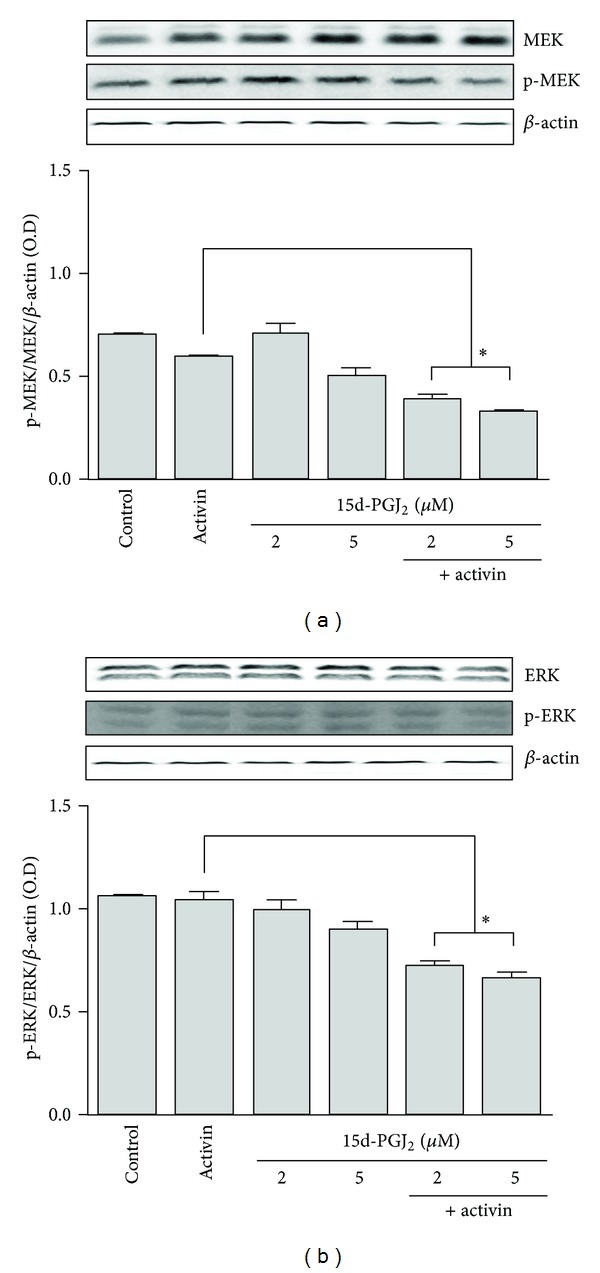
MEK and ERK protein expression in HepG2 cells. HepG2 cells were cultured with activin A (25 ng/mL) and 15d-PGJ_2_ (2 or 5 **μ**M) for 72 h. The cells were lysed, and 30 *µ*g of soluble protein was separated by performing electrophoresis on a 12% SDS-PAGE gel. Protein was detected by performing western blot analysis. The protein band intensities were quantified by using the *i*-image solution program, and the results are reported as the ratio of MEK (a) and ERK (b) to *β*-actin. The data represent the mean ± SD of 3 independent samples. *P < 0.05, comparison of stimulation with 15d-PGJ_2_ and activin A versus activin A stimulation alone.

HepG2 cells were treated with activin A and 15d-PGJ_2_ for 72 h. We determined the changes in the phosphorylation levels of key proteins in the MEK/ERK pathway by performing western blot analysis to evaluate the effects of activin A and 15d-PGJ_2_ on this pathway in HepG2 cells. Activin A decreased MEK or ERK activation compared to the control, whereas 15d-PGJ_2_ did not have an affect. When the cells were stimulated with both 15d-PGJ_2_ and activin A, 15d-PGJ_2_ suppressed activin-induced MEK and ERK activation compared to activin A stimulation alone (Figures 5(a) and 5(b)).

## 4. Discussion

The maximal response was approximately 40% of the control values and no further suppression in proliferation was seen following activin A exposure (5–50 ng/mL) [16]. A recent study showed that 15d-PGJ_2_ did not influence the HepG2 cell proliferation rate at low concentrations [17]. We investigated the effects of activin A and 15d-PGJ_2_ on HepG2 cell proliferation. Activin A (25 ng/mL) inhibited HepG2 cell proliferation, whereas 15d-PGJ_2_ (2 *μ*M and 5 *μ*M) had no effect. Our results are consistent with previous studies, which demonstrated that activin A suppresses cell proliferation, whereas 15d-PGJ_2_ has no effect.

We found that activin A increased the expression levels of most ActR and Smad transcripts compared to the control with the exception of ActR IIA mRNA levels, which were decreased, and Smad2 and Smad4 mRNA levels, which remained unchanged. 15d-PGJ_2_ stimulation decreased both ActR and Smad mRNA expression. However, costimulation with 15d-PGJ_2_ and activin A suppressed both activin-induced ActR and Smad mRNA expression in the HepG2 cells. These results suggest that activin and 15d-PGJ_2_ have differential regulatory effects at the transcriptional level. 15d-PGJ_2_ might inhibit Smad2 translocation through the PPAR*γ*/TGF-*β*/Smad2 pathway. The PPAR*γ* agonist 15d-PGJ_2_ inhibits the TGF*β*-induced connective tissue growth factor expression in human aortic smooth muscle cells [18]. In this study, 15d-PGJ_2_ inhibited activin-induced ActR and Smad expression, which suggests that 15d-PGJ_2_ ligands play important roles in regulating activin signaling pathways. The effects of PPAR*γ* on activin signaling have not been reported. Therefore, further research is necessary to evaluate this concept.

IL-6 stimulates the HepG2 production of fibrinogen in a concentration-responsive manner, whereas activin suppresses this IL-6 mediated activity [19]. 15d-PGJ_2_ plays a critical role in mediating IL-8 up-regulation, either via PPAR*γ* activation or via other mechanisms. PPAR*γ* agonists (15d-PGJ_2_ and troglitazone) inhibit the TGF*β*1-induced expression of chemokines in human tubular epithelial cells [20]. 15d-PGJ_2_ attenuates the NF-*κ*B-mediated transcriptional activation of many proinflammatory genes through PPAR*γ*-dependent and PPAR*γ*-independent mechanisms [21]. One of the most important transcription factors, which regulates the expression of IL-8, is NF-*κ*B [22]. We found that activin A and 15d-PGJ_2_ stimulation decreased IL-6 production and enhanced IL-8 production compared to the control. Activin A increased NF-*κ*B p65 activity, while 15d-PGJ_2_ did not affect NF-*κ*B p65 activity. However, costimulation with 15d-PGJ_2_ and activin A down-regulated activin-induced IL-6 production, up-regulated activin-induced IL-8 production, and inhibited activin A-induced NF-*κ*B p65 activity and MAPK signaling. Taken together, our results suggest that 15d-PGJ_2_ down- and up-regulates the production of activin-induced IL-6 and IL-8, respectively, by inhibiting the NF-*κ*B signaling pathway. TGF-*β* inhibits chemokine expression through a Smad-related pathway [23]. NF-*κ*B has been shown to suppress the TGF-*β*/Smad pathway through the transcriptional activation of Smad7, which is an inhibitory Smad [24]. Our results similarly suggest that chemokine expression in HepG2 cells is mediated by the Smad and NF-*κ*B signaling pathways. 15d-PGJ_2_ up-regulates IL-8 expression through the ERK1/2 pathway [15], and 15d-PGJ_2_-mediated IL-8 up-regulation is related to the NF-*κ*B and MAPK signaling pathways. 

## 5. Conclusions

15d-PGJ_2_ suppresses activin-induced ActR and Smad expression, down-regulates IL-6 production, and up-regulates IL-8 production, due to NF-*κ*B inhibition as well as to the negative regulation of MAPK activation in HepG2 cells. Regulation of ActR and Smad transcript expression and cytokine production involves NF-*κ*B and the MAPK pathway via interacting with 15d-PGJ_2_/activin/Smad signaling. However, the exact mechanisms that are involved in these regulatory pathways remain to be elucidated. 

## Abbreviations

15d-PGJ_2_: 15-Deoxy-Δ^12,14^-prostaglandin J_2_
ActR: Activin receptor
NF-*κ*B: Nuclear factor-*κ*B
MAPK: Mitogen-activated protein kinase
TGF-*β*: Transforming growth factor-*β*
PPAR*γ*: Peroxisome proliferator-activated receptor gamma
IL: Interleukin.
